# The Histone Modifications of Neuronal Plasticity

**DOI:** 10.1155/2021/6690523

**Published:** 2021-02-11

**Authors:** Huixia Geng, Hongyang Chen, Haiying Wang, Lai Wang

**Affiliations:** ^1^Institute of Chronic Disease Risks Assessment, School of Nursing and Health Sciences, Henan University, Kaifeng, 475004 Henan Province, China; ^2^College of Life Science, Henan University, Kaifeng, 475004 Henan Province, China

## Abstract

Nucleosomes composed of histone octamer and DNA are the basic structural unit in the eukaryote chromosome. Under the stimulation of various factors, histones will undergo posttranslational modifications such as methylation, phosphorylation, acetylation, and ubiquitination, which change the three-dimensional structure of chromosomes and affect gene expression. Therefore, the combination of different states of histone modifications modulates gene expression is called histone code. The formation of learning and memory is one of the most important mechanisms for animals to adapt to environmental changes. A large number of studies have shown that histone codes are involved in the formation and consolidation of learning and memory. Here, we review the most recent literature of histone modification in regulating neurogenesis, dendritic spine dynamic, synapse formation, and synaptic plasticity.

## 1. Introduction

Histone modification, as a precise regulation of gene expression to make cells adapt to changes in the environment, plays an important role in neuronal development, plasticity, and behavioral memory. Histone posttranslational modifications affect the spatial structure of chromatin, modulate the transcript and expression of genes, and change biological functions. Therefore, the combination of these histone modification patterns is called “histone codes” [1]. Accumulated evidence has shown that histone modification-mediated chromatin structure remodeling promotes the formation of excitatory synapses and hippocampal-dependent long-term memory. The most important forms of synaptic plasticity are long-term potentiation (LTP) or long-term depression (LTD). These two distinct types of synaptic plasticity reflect the increase and decrease of synaptic transmission efficiency, respectively, and extensive research has been conducted in the field of learning and memory.

## 2. Histone Modification

The genome of eukaryotes exists in the nucleus in the form of chromosome, which is composed of nucleosomes, which are the basic structure unit of chromatin, and packed with DNA. Two copies of histones H2A, H2B, H3, and H4 form an octamer core, and the approximately 147 base pair DNA is wrapped around the octamer core. Each nucleosome is linked by histone H1 to form higher-order chromatin fibre with a diameter of 30 nm [2, 3]. Increasing evidence has confirmed that at least 12 types of specific modifications occur to the N-terminal amino acid residues of histones, which affect the nucleosomes bind to DNA and the three-dimensional structure of chromosomes, and regulate gene expression. The patterns of histone modification are the following way: acetylation (lysine), methylation (lysine and arginine), phosphorylation (serine and threonine), sumoylation (lysine), ubiquitylation (lysine), ADP ribosylation, butyrylation, citrullination, crotonylation, formylation, proline isomerization, propionylation, serotonylation, and dopaminylation (glutamine) [4, 5]. The nomenclature of histone modification is as follows, such as H3K9me3, H3 refers to the core histone protein, K refers to the amino acid, the number 9 indicates the position of lysine residue from the N-terminal end of the amino acid tail of histone protein, and me3 refers to the type of modification on the lysine residue [6]. These different combinations and patterns of histone modifications are known as histone code [7].

The different amino acid residues at the N-terminal and C-terminal of these histones will undergo various posttranslational modifications, such as acetylation and methylation at lysine (K) or arginine (R) residues and phosphorylation at serine (S) or threonine (T) residues [8]. The histone acetylation modification is an important histone modification type, which means an increase in gene transcription activity and an epigenetic mark associated with dynamic chromatin. Histone acetyltransferases (HATs) catalyze the transfer of the acetyl group of acetyl-CoA molecule to lysine residues within the histone tails, while histone deacetylases (HDACs) remove these modifications. H3K27ac is related to active gene enhancers, and H4 acetylation is often found in the promoter region and bodies of activated genes. Therefore, histone acetylation can be used to compare gene transcript activity, especially H3 acetylation (H3ac) [9].

A number of amino acid residues in histones can be methylated, and the different methylation types show multiple valence states such as monomethylation (me 1), dimethylation (me 2), and trimethylation (me 3) forms. Therefore, histone methylation pattern changes may either promote gene expression or inhibit gene expression. Methylation of lysine 4 on histone 3 (H3K4) is one of the most studied modifications, with its trimethylated form (H3K4me3) enriched at transcriptional start sites (TSSs) of actively transcribed genes [10]. Histone methyltransferases (HMTs) and demethylases (HDM) are involved in the methylation modification process of histones. Histone methylation modification always occurs on both lysine (K) and arginine (R) side-chain groups of H3 and H4 histone tails. Trimethylaton of lysine 9 on histone 3 (H3K9me3) is often associated with chromatin superaggregation and heterochromatin, and the state of H3K27me3 affects the conversion from heterochromatin to euchromatin [11, 12]. Different modification types of histones also influence each other and together regulate the expression of specific genes. For example, the increase in H3K4me3 increases H3K9Ac, which indicates the mutual regulation function between histone modification and the complexity of gene expression regulation [13].

## 3. The Effect of Histone Modifications in Neuroplasticity

**Figure 1:**
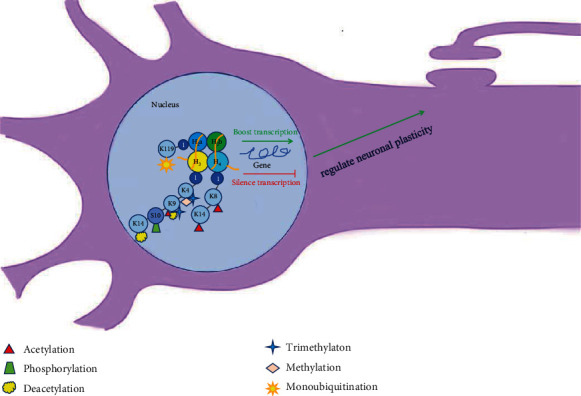
Histone modification mediates neuronal plasticity.

The brain makes higher animals more adaptable to environmental changes through the process of learning and memory. The formation of learning and memory is inseparable from the synaptic connections between neurons. Histone acetylation and deacetylation modification modulate the chromatin structure to regulate the synaptic connectivity and memory storage-related gene expression. On the other hand, the histone methylation modification states of the promoter region of the gene that controls the synaptic function have also changed, such as the increase of H3K4me3 [14]. More importantly, histone deacetylases 2 (HDAC 2) are enriched at the promoter regions or gene bodies of several neuroplasticity-associated genes, such as BDNF IV, activity-regulated cytoskeleton-associated protein (Arc), Cdk5, Egr1, Homer1, Gria1 and 2, neurofilament light protein (Nfl), N-methyl-D-aspartate receptor subunit 2B (NR2B), synaptophysin (Syn), and synaptotagmin (Syt) [15]. It can be seen that histone modification is involved in the formation of neuronal plasticity and memory formation and consolidation (Table 1 and Figure 1).

In cell nucleus, the histones at gene promoters related to neuroplasticity are modified by acetylation, phosphorylation, methylation, and other modifications, which alter the affinity of histones with these gene promoters. Thereby, it would activate or inhibit the transcriptional activity of these genes and regulate neuroplasticity.

### 3.1. Neurogenesis

The marks of histone methylation affect the pluripotency of neural progenitor cell (NPC) and determine their neural lineage specification and neuronal differentiation [16]. Polycomb group proteins (PcGs) and trithorax group proteins (TrxGs) antagonistically affect gene expression by histone methylation and demethylation to regulate neuronal differentiation and development. The PcGs are mainly divided into two types of multiprotein complexes, polycomb repressive complexes 1 (PRC1) and 2 (PRC2) [17]. In the central nervous system, the H3K27me3 repressive marks are removed by the histone demethylase Jmjd3 during the differentiation of embryonic stem cells (ESCs) into neural progenitor cell (NPCs) [18]. However, PcGs can prevent the differentiation of ESCs into NPCs by increasing the level of H3K27me3 at neuronal-specific genes such as *Ngns*, *Pax6*, and *Sox1*. In NPCs, the deletion of the subunit Ezh2 of PRC2 signally promotes neurogenesis and neuronal differentiation. In addition, Ezh2 gene silencing is also related to nerve migration [19–21].

RING1B, the core catalytic subunit of PRC1, catalyze H2AK119 monoubiquitination modification at promoters of neuronal-specific genes, such as *Tuj1*, *Ncan*, and *Nestin* to induce human embryonic stem cells (hESCs) and human pluripotent stem cells (hiPSCs) towards neural progenitor differentiation [22]. A maternal low choline diet significantly decreases H3K27me3 levels at the Toll-like receptor 4 (Tlr4) and increases Tlr4 expression, which mediate neuronal differentiation in fetal mouse neural progenitor cells [23]. During neurogenesis, the methylation of H3K4 and H3K36 is often used to indicate increased gene transcription activity, and the removal of H3K4me3 inhibits the expression of neurogenesis-related genes [24].

### 3.2. Axon Formation

The next-generation sequencing method has confirmed that there are multiple mutations in genes that encode chromatin structure regulators in the neurodevelopmental and psychiatric disorders, such as members of the lysine methyltransferase 2 (KMT2A/C/D) and lysine demethylase 5 (KDM5A/B/C) families. KMT2 catalyzes the di- and trimethylation of H3K4, while KDM5 executes H3K4 demethylation [25–27]. These results suggest that the methylation of H3K4 is very important for the neuronal development and function in the central nervous system [28, 29]. RBR-2, the sole homolog of the KDM5 family of H3K4me3/2 demethylases in *Caenorhabditis elegans*, controls the axon guidance and growth by regulating the expression of the actin regulator *wsp-1*. Knockout of the *rbp-2* gene increases the level of H3K4me3 at the transcription promoter site of the *wsp-1* gene, resulting in the abnormal transcription and high expression of *wsp-1*, leading to abnormal formation of axon guidance, and similar results as mutations in H3K4 methyltransferase KMT2F/G (SETD1A/B) genes [30, 31].

In addition to histone methylation regulating axon growth, histone acetylation also regulates axon regeneration and growth. Some research results in the lamprey spinal cord injury model showed that the expression of HDAC1 was upregulated in regenerated neurons, while the expression of HDAC1 did not change in nonregenerating neurons. These results suggest that HDAC1 is involved in neuron regeneration and axon growth [32].

### 3.3. Density and Morphology of Dendritic Spines

Many years of research has shown that the phosphorylation of the histone H3 protein induces the transformation of chromatin from a heterochromatin state to a euchromatin state, increases the transcriptional activity of genes, regulates the formation and maintenance of dendritic spines, and participates in the process of environmental learning and cognitive formation [33, 34].

The p90 ribosomal S6 kinase (p90RSK) was a protein shuttling from the cytosol to the nucleus, which has multiple functions. In quiescent cells, p90RSK forms a complex with the upstream regulatory molecule ERK1/2 in the cytoplasm. Once the cell is activated by growth factors and ROS, p90RSK would translocate to the nucleus and regulate gene expression through prompting I*κ*-B, c-Fos, Nur77, and cAMP-response element-binding protein and other downstream molecule phosphorylation [35]. NMDA receptor activation is crucial for long-term memory. Glycine activates the NMDA receptor of cortical neurons *in vitro*, and p90RSK accumulates in the nucleus in a time-dependent manner, phosphorylate histone H3 at serine 10 (H3S10p), changes the structure of dendritic spines, and regulates the remodeling of dendritic spines [36].

Histone acetylation and methylation modifications also regulate dendritic spine morphogenesis and increase in density. Acute ethanol exposure reduced the activity of HDAC and the expression level of HDAC2 protein in the central nucleus of the amygdala of rat, increased the histone acetylation status of brain-derived neurotrophic factor (BDNF), and activity-regulated cytoskeleton-associated protein (Arc) genes, which attenuated anxiety-like behaviors [37]. ANP32A is one of the most important members of histone acetyltransferase inhibitor, which is abnormally highly expressed in the brains of Alzheimer's disease (AD) patients and model mice. The hippocampal infusion of lenti-siANP32A reduces the ANP32A expression in AD model mice with human tau transgenic, which increases the acetylation of histones in the neuron, promotes the formation of dendritic spines, and restores the learning and memory ability of mice [38]. Histone deacetylase inhibitors increase the number of dendritic spines, enhance synaptic plasticity, and improve memory in young mice [39].

Inhibition of methyltransferase SUV39H1 activity decreases the level of H3K9me3 in the hippocampus of aged mice, which increases BDNF expression levels, and the spine density of thin and stubby. Furthermore, the inhibition of SUV39H1 reversed the deficits in hippocampal memory through enhancing surface GluR1 levels in hippocampal synaptosomes and facilitating synaptic plasticity [40]. The inhibition of H3K4 histone methyltransferase (HMT) activity decreased the level of H3K4me1, H3K4me3, and H3K9Ac, which increased the density of dendritic spine densities in SY5Y cells [41]. UTX, a histone demethylase, could remove the repressive trimethylation of histone H3 lysine 27 (H3K27me3). UTX regulate the expression of a subset of genes that are involved in the regulation of dendritic morphology, synaptic transmission through modulating expression of neurotransmitter 5-hydroxytryptamine receptor 5B (Htr5b) in mouse hippocampal neurons [42].

### 3.4. Synaptic Plasticity

The BDNF is an important neurotrophin in neuronal survival and growth, which is involved in synaptogenesis, synaptic plasticity, and memory consolidation. Under the different environmental stimuli, the expression of the BDNF gene is regulated by epigenetic mechanisms such as histone modification, DNA methylation, and microRNA machineries [43]. HDAC inhibitors increase the acetylation of the RNA polymerase II at the promoter 3 BDNF gene and histone H4, which enhance BDNF transcription and expression [44]. Cultured rat hippocampal slices in vitro, BDNF treatment increases the density of mature spines (type-I stubby and type-II mushroom) and immature type-III thin spines, enhancing the release of excitatory neurotransmitters and excitatory postsynaptic current (EPSC) in hippocampal slice cultures, while the HDAC inhibitor TSA (trichostatin-A, TSA) reverses this process. Mechanism studies have shown that H3 at lysines 9 and 14 acetylation states participate in this process [45]. The treatment of TSA promotes the BDNF and activity-regulated cytoskeleton-associated protein (Arc) gene expression in the amygdala of rats, increases the dendritic spine density, facilitates synaptic plasticity, and attenuates anxiety-like behaviors of rats [46].

Proper histone acetylation level at the promoter region of the BDNF gene affects LTP and long-term memory. The acetylation of histones H3 and H4 at the activity-dependent BDNF promoters I and IV upregulates the transcription and expression of BDNF in the hippocampus, then enhances the consolidation of fear memory in rats [47]. The expression level of BDNF is decreased in AD patients and mouse model [48, 49]. The sulforaphane, a universal inhibitor of HDAC, is extracted from the hydrolysis product of glucoraphanin present in Brassica vegetables. Sulforaphane inhibited HDAC activity and increased global acetylation states of histone 3 (H3) and H4 in primary cortical neurons. Additional analyses show that sulforaphane enhanced BDNF expression and increased levels of neuronal and synaptic molecules such as microtubule-associated protein 2 (MAP2), synaptophysin, and PSD-95, as well as elevated levels of synaptic TrkB signaling pathway components in primary cortical neurons and 3 × Tg-AD mice [50]. Microglia is the main form of immune defense in the central nervous system and also has been shown to play a significant role in the synaptic plasticity in neurons. The histone sumoylation modification regulates the expression of phosphatidylinositol 3-kinase (PI3K), the phosphorylation of AKT and CREB, and the expression of BDNF. The ablation or disruption of microglial function abolishes long-term potentiation (LTP) and reduces synaptic plasticity in rat hippocampal slices [51].

In addition to affecting BDNF gene expression to modulate synaptic plasticity, histone modification also facilitates synaptic plasticity by affecting the expression of other genes, which affects cognition and memory functions. The CAAT box enhancer binding protein (CBP), a histone acetylase, could induce gene expression for the growth of new synapses and for increasing synaptic strength through acetylating lysine residues on core histones. CREB1 recruits CBP into the gene promoter region, and acetylase lysine 8 (K8) of histone H4 and lysine 14 (K14) of histone H3, which form long-term synaptic plasticity with increased synapse strength called long-term facilitation. On the contrary, FMRFamide, an inhibitory transmitter, promotes recruitment HDAD5 and reduces the acetylation of K8 of histone H4 and C/EBP gene expression, which switch the synaptic plasticity to long-term depression [52]. Tip 60, a multifunctional HAT, could modulate the expression of neurogenesis-related genes through epigenetic methods. Targeted loss of Tip60 HAT activity causes thinner and shorter axonal lobes in mushroom body (MB) in the central nervous system of *Drosophila* and defects in immediate-recall memory, while increasing Tip60 HAT levels reverse these process and facilitate immediate-recall memory and cognitive function [53]. K-Acetyltransferase 2a (Kat2a) is a multifunctional histone acetyltransferases (HATs) that has the highest level expression in the hippocampal CA1 region. Kat2a regulates the expression of genes related to the hippocampal gene network linked to the neuroactive receptor by activating the NF-*κ*B signaling pathway, thereby participating in hippocampal synaptic plasticity and long-term memory consolidation [54]. The neuron-specific overexpression of HDAC2 but not that of HDAC1 would reduce dendritic spine density, number of synapses, synaptic plasticity, and memory formation [55].

G9a/G9a-like protein (G9a/GLP), a lysine dimethyltransferase, is an essential role for the formation of the histone H3 lysine 9 dimethylation (H3K9me2). The inhibition of G9a/GLP activity in the entorhinal cortex (EC) enhances H3K9me2 in the area CA1, leading to the silencing of nonmemory permissive gene COMT in the hippocampus, promoting synaptic plasticity, and is conducive to the formation and consolidation of long-term memory. However, the mechanism by which G9a/GLP activity mediates the formation and consolidation of long-term memory (LTM) is different in the hippocampus and entorhinal cortex of SD rats [56]. *α*-Synuclein (*α*S) is mostly localized within synapses and linked to Parkinson's disease (PD). The expression level of euchromatic histone lysine N-methyltransferase 2 (EHMT2) was increased in *α*S-induced SH-SY5Y cells, which enhanced H3K9 methylation (H3K9me2) and the synaptosomal-associated protein SNAP25 expression. Thus, *α*S overexpression enhances synaptic vesicle fusion events and synaptic plasticity through increasing *ΕΗΜΤ*2 expression to elevate H3K9me2 at the SNAP25 promoter [57]. The cannabis consumption leads to a large number of mental illnesses among adolescents. The *Δ*9-tetrahydrocannabinol (THC) exposure significantly induce the expression of histone methyltransferase (Suv39H1) and enhance 3K9me3 in the prefrontal cortex of female rats after adolescent. Moreover, the *Δ*9-tetrahydrocannabinol (THC) exposure downregulates the expression of synaptic plasticity genes, reduces the time for rats to recognize new objects in object recognition experiment (NOR), and destroys cognitive function [58].

## 4. Summary and Outlook

A large number of research studies have shown that the synaptic plasticity-associated gene expression by neuroepigenetic regulation is essential for the formation and consolidation of learning and memory, and histone modification is one of the most important ways of neuroepigenetic regulation. Under the stimulation of different factors, histones in neurons will undergo various types of chemical modification, such as methylation/demethylation, acetylation/deacetylation, and phosphorylation/dephosphorylation, which could change the three-dimensional structure of chromosomes in the promoter region of synaptic plasticity-related genes and regulate the transcriptional activity of these genes. In the process of neurological disorder, the degradation of dendrites and axons and the reduction of synaptic plasticity are the early morphological pathological changes of the disease. At present, numerous studies on the effect of histone modification inhibitors on synaptic plasticity have been extensively carried out in neurological disorder animal models and achieved meaningful research results. However, in the process of applying histone modification inhibitors to affect synaptic plasticity, how to ensure that the inhibitor can accurately enter the nervous system instead of spreading to the whole body is a problem that must be considered. Secondly, many types of histone modifications are involved in the regulation of synaptic plasticity, but how to determine a certain type of modification is the dominant factor in the regulation of synaptic plasticity in a specific condition, which is also a consideration important factor for the application of histone inhibitors to the prevention and treatment of neurological diseases.

## Figures and Tables

**Table 1:** The histone modification in neuronal plasticity.

Histone	Amino acid	Modification	Method	Impact in neuronal plasticity	References
H2A	K119	Monoubiquitination	The human embryonic stem cells and human pluripotent stem cells	Induce neural progenitor differentiation	Desai et al., 2020 [22]

H3	K9	Deacetylation	Rats	Decreased dendritic spine density	Moonat et al., 2013 [37]
	S10	Phosphorylation	The cortical neuron from Sprague-Dawley rat	Remodel dendritic spine morphology	VanLeeuwen et al., 2014 [36]
	K9, K14	Deacetylation	The hippocampal slices of rat	Increase dendritic spine density and excitatory quantal transmitter release	Calfa et al., 2012 [44]
	K9	Trimethylation	Female Sprague-Dawley rat	Impact the expression of genes with synaptic plasticity	Prini et al., 2017 [57]
	K9	Acetylation	SY5Y cell	Increase dendritic spine density	Al Sayed et al., 2019 [41]
	K4	Monomethylation and trimethylation	SY5Y cell	Increase dendritic spine density	Al Sayed et al., 2019 [41]

H4	K14	Acetylation	Aplysia cell culture	Induce synapse-specific long-term facilitation	Guan et al., 2002 [51]
	K8	Acetylation	Aplysia cell culture	Induce synapse-specific long-term facilitation	Guan et al., 2002 [51]

## Abbreviations

LTP: Long-term potentiation
LTD: Long-term depression
HATs: Histone acetyltransferases
HDACs: Histone deacetylases
HMT: Histone methyltransferases
Syn: Synaptophysin
Syt: Synaptotagmin
p90RSK: p90 ribosomal S6 kinase
ROS: Reactive oxygen species
NMDA: N-Methyl-D-aspartate
BDNF: Brain-derived neurotrophic factor
AD: Alzheimer's disease
EPSC: Excitatory postsynaptic current
MAP2: Microtubule-associated protein 2
CBP: CAAT box enhancer binding protein
PD: Parkinson's disease
NOR: Object recognition experiment
ESC: Embryonic stem cells
NPC: Neural progenitor cell.
